# Assessment of knowledge, attitude, and practice toward diabetic retinopathy among type 2 diabetic patients attending primary health care clinics in Riyadh, Saudi Arabia

**DOI:** 10.1097/MS9.0000000000004679

**Published:** 2026-07-31

**Authors:** Khaled Almadani, Fahad Almujarri, Feras Eid, Abdulrahman Alshalawi, Musaad Alsiyat, Rayyan Alturkistani

**Affiliations:** a Ambulatory Care Services, National Guard Health Affairs, Riyadh, Saudi Arabia; b College of Medicine, King Saud bin Abdulaziz University for Health Sciences, Riyadh, Saudi Arabia

**Keywords:** diabetic retinopathy, eye screening, knowledge, primary healthcare, Saudi Arabia, type 2 diabetes

## Abstract

**Background:**

Diabetic retinopathy (DR) is a leading cause of preventable vision loss among individuals with type 2 diabetes mellitus (T2DM). Despite the availability of effective screening and treatment, adherence to regular eye examinations remains suboptimal in Saudi Arabia. Understanding patients’ knowledge, attitudes, and practices (KAP) toward DR is crucial for developing targeted interventions within primary healthcare.

**Purpose:**

To evaluate the KAP regarding DR among patients with T2DM attending primary healthcare clinics in Riyadh, Saudi Arabia.

**Methods:**

A cross-sectional study was conducted among 400 patients with T2DM using a validated, structured questionnaire. Descriptive statistics, Mann–Whitney *U*, Kruskal–Wallis tests, and Spearman’s rank correlation were applied to examine KAP scores and their associations with sociodemographic and clinical variables.

**Results:**

Only 28.5% of participants had undergone DR screening within the past year, and just 10.5% recalled receiving physician advice to do so. Knowledge scores were moderate (mean = 1.77 ± 0.23), but attitudes were largely negative (mean = 1.20 ± 0.20). Screening practices were suboptimal (mean practice score = 1.53 ± 0.29). Education level, gender, occupation, and duration of diabetes were significantly associated with KAP domains (*P* < 0.05).

**Conclusion:**

Despite moderate awareness of DR, most participants demonstrated poor attitudes and limited adherence to recommended screening. Our study emphasized the physician-related barriers and highlighted the importance of culturally tailored education and integrated primary care approaches to improve DR screening uptake.

## Introduction

Diabetic retinopathy (DR) is a leading microvascular complication of diabetes and a primary cause of visual impairment among working-age adults worldwide. The global rise in diabetes prevalence, particularly in low- and middle-income countries, has resulted in an increasing burden of DR. Locally, the International Diabetes Federation stated that 23.1% of adults in Saudi Arabia are estimated to have diabetes, which places a significant strain on the national healthcare system and contributes to a growing incidence of diabetic eye disease^[^1^]^.

HIGHLIGHTS

- Most patients had a moderate knowledge about diabetic retinopathy.
- Attitude and practices toward eye screening were suboptimal.
- Awareness was significantly associated with education and the duration of diabetes.
- Only a third of participants had regular eye check-ups.
- Findings support the need for referral systems and public health education.

Early DR often presents without noticeable symptoms, making regular retinal screening critical for timely detection and intervention. Evidence indicates that early identification and treatment can reduce the risk of severe vision loss by up to 95%^[^2^]^. However, despite the availability of screening services, uptake remains inadequate. For example, a local study reported that only 27.5% of diabetic patients underwent annual retinal screening^[^3^]^, a trend mirrored in other regions of the Kingdom^[^4^]^. Similarly, a national Saudi study highlighted that 29% of diabetic patients had never undergone an eye examination^[^5^]^.

Barriers to screening include limited awareness, misperceptions about the asymptomatic nature of DR, and insufficient physician recommendations^[^5^]^. Primary healthcare providers play a pivotal role in patient education and early referral for ophthalmologic assessment, yet few studies have explored the knowledge, attitudes, and practices (KAP) of T2DM patients within Saudi Arabia’s primary care context. Understanding these factors is essential for designing effective public health interventions. As Riyadh represents one of the most populous and demographically diverse regions of Saudi Arabia, it was selected as a representative setting for this cross-sectional study.

This study aims to evaluate the KAP related to DR among patients with T2DM attending primary healthcare clinics in Riyadh and to identify sociodemographic determinants associated with suboptimal eye care behaviors. This manuscript has been reported as per the STROCSS 2025 criteria^[^6^]^.

## Methods

### Study design and setting

A descriptive cross-sectional study was conducted between January and March 2024 at a primary healthcare center in Riyadh, Saudi Arabia. The study aimed to assess KAP regarding DR among patients diagnosed with type 2 diabetes mellitus (T2DM).

### Study population

A total of 400 adult patients with a confirmed diagnosis of T2DM were recruited using convenience sampling during routine visits to the clinic. Because a convenience sampling technique was employed, the findings may not be generalizable to all individuals with T2DM in Saudi Arabia.

Inclusion criteria were:

- Age ≥18 years.
- Confirmed diagnosis of T2DM for at least 1 year.
- Ability to provide informed consent and participate in a structured interview.

Exclusion criteria included:

- Diagnosis of type 1 diabetes or gestational diabetes.
- Cognitive impairment, visual or hearing disabilities that interfered with questionnaire completion.

Participation was voluntary, and patients could withdraw at any stage without consequence.

The sample size of 400 was estimated using Cochran’s formula for cross-sectional studies, assuming a 50% response distribution, a 5% margin of error, and a 95% confidence level, based on previous regional KAP research.

### Data collection instrument

Data were collected using a structured, interviewer-administered questionnaire adapted from previously validated tools used in diabetic complications research in Saudi Arabia and other Middle Eastern populations^[^2,7,8^]^. Interviewers read each questionnaire item verbatim to the participant and recorded the chosen response accordingly. No additional explanation or interpretation was provided, ensuring consistency and minimizing interviewer bias.

The original instrument was developed in English, translated into Arabic, and back-translated by a bilingual expert to ensure semantic and conceptual equivalence.

A pilot test was conducted with 20 T2DM patients to evaluate content clarity, internal consistency, and cultural relevance. Based on feedback, minor linguistic adjustments were made. Pilot data were excluded from the final analysis. The final version demonstrated acceptable internal consistency, with a Cronbach’s alpha of 0.79 across all domains.

### Questionnaire structure and scoring

The final questionnaire consisted of four sections:

Sociodemographic data (e.g., age, gender, education, and occupation)

Clinical characteristics (e.g., duration of diabetes and comorbidities)

Knowledge of DR (e.g., risk factors, symptoms, and screening).

Attitude and practice toward DR screening and management: Responses in the KAP sections were scored as follows:

Correct/positive: 2 points

Incorrect/negative: 1 point

“I do not know”: 0 points

Total scores for each domain were converted into percentages and categorized as:

Good: ≥ 75%

Moderate: 50–74%

Poor: <50%.

### Statistical analysis

All statistical analyses were performed using IBM SPSS Statistics for Windows, version 29 (IBM Corp., Armonk, NY, USA). Descriptive statistics, including frequencies, percentages, means, and standard deviations, were used to summarize participants’ characteristics and KAP scores.

Owing to the non-normal distribution of data, non-parametric tests were employed to assess the bivariate associations between sociodemographic/clinical factors and KAP scores as follows:

Mann–Whitney *U* test for binary variables.

Kruskal–Wallis test for categorical variables with more than two groups.

Spearman’s rank correlation for continuous variables (e.g., age). A *P*-value of <0.05 was considered statistically significant.

## Results

### Participant demographics

A total of 400 patients with T2DM participated in the study. The mean age was 58.0 years (standred deviation [SD] ± 12). The majority were male (*n* = 349, 87.3%). Most participants had completed either college (42.8%) or high school (37.5%). Regarding occupational status, 50.5% were retired, and 37.8% were employed. Nearly half of the respondents (48.8%) had been diagnosed with diabetes for more than 10 years. Common comorbidities included hypertension (41.0%), dyslipidemia (20.5%), and heart disease (14.8%) (Table 1).

**Table 1:** Baseline features of included participants.

Variables	Mean	Standard deviation	Count	Layer *N* (%)
Age	58	12			
Gender
Male			349	87.3
Female			51	12.8
Level of education
Uneducated			16	4.0
Elementary			26	6.5
Intermediate			37	9.3
High school			150	37.5
College and above			171	42.8
Occupation
Employed			151	37.8
Unemployed			47	11.8
Retired			202	50.5
Duration of DM
Less than 5 years			109	27.3
5–10 years			96	24.0
More than 10 years			195	48.8
Hypertension
Yes			164	41.0
No			236	59.0
Dyslipidemia
Yes			82	20.5
No			318	79.5
Heart disease
Yes			59	14.8
No			341	85.3

### Eye screening and physician advice

Only 28.5% (*n* = 114) of participants reported undergoing a diabetes-related eye examination within the past year. While 67.8% had seen an eye doctor for any eye-related complaint in the past year, only 34.0% had undergone retinal imaging specifically for diabetes screening. Notably, just 10.5% (*n* = 42) of patients recalled being advised by a physician to obtain annual eye check-ups. These findings reflect limited engagement with recommended DR screening protocols. The overall mean eye information score was 1.56 (SD ± 0.29), indicating a moderate level of awareness (Table 2).

**Table 2:** Information level of included participants.

	Count	Layer, *N* (%)	Mean	Standard deviation
Did you go to an eye doctor in the last 1 year for an eye check-up of diabetes?
Yes	114	28.5		
No	286	71.5		
I do not know	0	0.0		
Did you go to the eye doctor in the last 1 year for any other eye problem?
Yes	271	67.8		
No	129	32.3		
I do not know	0	0.0		
Was any photograph of the inside of my eye taken for diabetes last year?
Yes	136	34.0		
No	264	66.0		
I do not know	0	0.0		
Did your doctor advise you for an eye check-up at least once every year?
Yes	42	10.5		
No	358	89.5		
I do not know	0	0.0		
Have you undergone laser treatment for diabetes in the last 2 years?
Yes	314	78.5		
No	86	21.5		
I do not know	0	0.0		
Information score			1.56	0.29

### Knowledge of DR

Participants demonstrated limited knowledge regarding DR. Only 5.5% correctly identified vision loss as a potential complication of diabetes, and 4.3% recognized the importance of glycemic control in preventing eye damage. Awareness of asymptomatic early DR changes (38.5%) and the role of dilated retinal exams (43.0%) was modest. The mean knowledge score was 1.77 (SD ± 0.23), classifying overall knowledge as moderate (Table 3).

**Table 3:** Knowledge level of included participants.

	Count	Layer, *N* (%)	Mean	Standard deviation
A person with diabetes can lose his/her eyesight due to complications.
Yes	22	5.5		
No	378	94.5		
I do not know	0	0.0		
Control of blood sugar can help a diabetic to avoid/delay complications in the eye.
Yes	17	4.3		
No	383	95.8		
I do not know	0	0.0		
Early changes in the eye due to diabetes can be without defective vision.
Yes	154	38.5		
No	246	61.5		
I do not know	0	0.0		
Retina (shabakia) examination after putting eye drops to dilated pupil by expert and by using special equipment is essential for early detection of diabetic retinopathy.
Yes	172	43.0		
No	228	57.0		
I do not know	0	0.0		
Once vision is damaged in diabetes, it is often late to restore eyesight.				
Yes	108	27.0		
No	292	73.0		
I do not know	0	0.0		
Laser treatment delays serious damage due to diabetic retinopathy.				
Yes	164	41.0		
No	236	59.0		
I do not know	0	0.0		
Even after eye treatment for diabetic retinopathy, one should control diabetes.				
Yes	16	4.0		
No	384	96.0		
Otherwise, the vision can further deteriorate.				
Knowledge score			1.77	0.23

### Attitudes toward DR screening

Negative perceptions toward DR screening were prevalent. A majority (91.8%) agreed that frequent eye examinations were a waste of time. Only 44.0% believed that physicians are responsible for managing eye complications related to diabetes. Moreover, 89.5% doubted the effectiveness of DR treatment. The mean attitude score was 1.20 (SD ± 0.20), indicating generally poor attitudes toward screening and intervention (Table 4).

**Table 4:** Attitude level of included participants.

	Count	Layer, *N* (%)	Mean	Standard deviation
Frequent eyes check up by expert for diabetic retinopathy is waste of time for patient.
Yes	367	91.8		
No	33	8.3		
My physician is responsible for taking care of my diabetes including eye complication.
Yes	176	44.0		
No	224	56.0		
A diabetic patient should focus on complications of kidney and heart instead of eyes.
Yes	380	95.0		
No	20	5.0		
Treatment of diabetic retinopathy is not benefiting diabetic patients.
Yes	358	89.5		
No	42	10.5		
Attitude score			1.20	0.20

### Practices related to diabetes and eye care

Participants reported varying adherence to diabetes self-management behaviors. Regular blood pressure monitoring (65.0%) and weight tracking (64.3%) were common, but only 29.5% monitored their blood sugar regularly and 29.0% followed a diabetes-specific diet. Compliance with recommended annual DR screening was also low, with only 47.5% reporting adherence. The mean practice score was 1.53 (SD ± 0.29), reflecting a moderate level of engagement in preventive behaviors (Table 5).

**Table 5:** Practice level of included participants.

	Count	Layer, *N* (%)	Mean	Standard deviation
I take care of my diet as I am diabetic.
Yes	116	29.0		
No	284	71.0		
I do not know	0	0.0		
I check my blood sugar regularly.
Yes	118	29.5		
No	282	70.5		
I do not know	0	0.0		
I check my blood pressure regularly.
Yes	260	65.0		
No	140	35.0		
I do not know	0	0.0		
I check my weight regularly.
Yes	257	64.3		
No	143	35.8		
I do not know	0	0.0		
I visit my eye doctor for DR screening every year or more frequently if my eye doctor has advised.
Yes	190	47.5		
No	210	52.5		
I do not know	0	0.0		
Practice score			1.53	0.29

### Associations between demographics and KAP scores

Several statistically significant associations were observed between participant characteristics and KAP scores.

Respondents with higher education levels and those who were employed exhibited significantly greater knowledge scores compared to less educated or unemployed individuals (*P* < 0.05). In the same context, the eye information score was notably higher among males, participants with extended diabetes duration, and individuals with hypertension (*P* < 0.05).

In contrast, attitude scores were significantly influenced by education, employment status, duration of diabetes, and the presence of hypertension or dyslipidemia (*P* < 0.05).

Regarding practice scores, better adherence to recommended eye-care behaviors was linked to higher education levels and a history of hypertension or heart disease (*P* < 0.05) (Tables 6 and 7).

**Table 6:** Association of baseline features with information and knowledge scores of included participants.

Eye information knowledge				
	Mean	SD	*P*-value^M/K^	Mean	SD	*P*-value^M/K^
Gender
Male	1.55	0.29	0.022	1.78	0.22	0.034
Female	1.65	0.27		1.69	0.26	
Level of education
Uneducated	1.68	0.26	0.000	1.70	0.20	
Elementary	1.76	0.17		1.77	0.26	0.060
Intermediate	1.65	0.25		1.72	0.23	
High school	1.57	0.28		1.80	0.23	
College and	1.49	0.30		1.75	0.23	
above						
Occupation
Employed	1.49	0.29	0.001	1.78	0.22	
Unemployed	1.63	0.23		1.67	0.26	0.013
Retired	1.60	0.29		1.78	0.23	
Duration of DM
Less than 5	1.46	0.31	0.000	1.75	0.26	
years						0.570
5–10 years	1.53	0.30		1.75	0.22	
More than 10	1.63	0.25		1.78	0.22	
years						
Hypertension
Yes	1.63	0.27	0.000	1.75	0.22	
No	1.51	0.29		1.78	0.24	0.160
Dyslipidemia
Yes	1.57	0.27	0.823	1.76	0.21	
No	1.56	0.29		1.77	0.24	0.601
Heart disease
Yes	1.61	0.29	0.103	1.72	0.23	
No	1.55	0.29		1.77	0.23	0.080

M/K = Mann–Whitney *U* test or Kruskal–Wallis test.

**Table 7:** Association of baseline features with attitude and practices scores of included participants.

	Attitude			Practices		
	Mean	SD	*P*-value^M/K^	Mean	SD	*P*-value^M/K^
Gender						
Male	1.20	0.20	0.477	1.52	0.29	0.084
Female	1.21	0.17		1.59	0.27	
Level of education						
Uneducated	1.09	0.13	0.021	1.61	0.24	0.013
Elementary	1.21	0.24		1.51	0.31	
Intermediate	1.15	0.22		1.66	0.22	
High school	1.21	0.20		1.49	0.30	
College and above	1.21	0.19		1.53	0.29	
Occupation						
Employed	1.18	0.18	0.018	1.52	0.28	0.860
Unemployed	1.28	0.22		1.53	0.26	
Retired	1.20	0.20		1.54	0.30	
Duration of DM						
Less than 5 years	1.17	0.18	0.002	1.50	0.29	0.367
5–10 years	1.16	0.17		1.55	0.28	
More than 10 years	1.24	0.21		1.54	0.28	
Hypertension						
Yes	1.17	0.19	0.012	1.59	0.29	0.000
No	1.22	0.20		1.48	0.28	
Dyslipidemia						
Yes	1.16	0.21	0.011	1.58	0.28	0.050
No	1.21	0.19		1.52	0.29	
Heart disease						
Yes	1.19	0.22	0.377	1.62	0.28	0.009
No	1.20	0.19		1.51	0.28	

BM, diabets mellitis; M/K = Mann–Whitney *U* test or Kruskal–Wallis test; SD standred deviation.

### Correlation with age

Spearman’s correlation analysis indicated a significant negative correlation between age and both the eye information score (*P* = 0.026) and the attitude score (*P* = 0.002), suggesting that older participants were less informed and more likely to hold unfavorable views. No significant correlation was observed between age and the knowledge or practice scores (Table 8).

**Table 8:** Association among age, eye information, knowledge, attitude, and practices with different scores of the included participants.

	Eye information	Knowledge	Attitude	Practices
	*P*-value*	*P*-value*	*P*-value*	*P*-value*
Age	0.026	0.502	0.002	0.979

^*^ Spearman correlation.

## Discussion

This study highlights persistent deficiencies in the KAP regarding DR among patients with T2DM attending primary healthcare clinics in Riyadh, Saudi Arabia. Although moderate knowledge scores were observed, the widespread negative attitudes and poor engagement with DR screening protocols are concerning and warrant public health attention.

The limited awareness of DR’s asymptomatic progression and the low recognition of its link to vision loss echo findings from similar studies across the Gulf region and Asia, where cultural, systemic, and educational barriers impact screening behavior^[^4,5^]^. For example, only 5.5% of patients in this study recognized that diabetes can cause vision loss, and fewer than one-third underwent an eye examination in the past year, despite national guidelines recommending annual screenings.

Perhaps the most striking finding was that only 10.5% of participants recalled receiving physician advice for DR screening, a critical missed opportunity for preventive care. This aligns with previous reports that cite insufficient physician–patient communication as a significant barrier to screening uptake^[^5,9^]^. Physician recommendation is among the strongest predictors of compliance with DR screening^[^10^]^, highlighting the urgent need to integrate eye health education into primary diabetes care workflows. The low rate of physician-initiated DR screening may reflect limited consultation time, lack of systematic referral prompts, and the absence of integrated ophthalmology pathways in primary care. Additionally, cultural fatalism and low health literacy may foster the perception that vision loss is an inevitable outcome of diabetes, thereby discouraging preventive eye^[^5^]^.

Negative attitudes were pervasive. Over 90% of respondents viewed routine eye check-ups as unnecessary, and nearly 90% doubted the benefits of DR treatment. These beliefs may stem from low health literacy, fatalistic health attitudes, or past healthcare experiences. Our results also showed that unfavorable attitudes were more common among older patients, those with less education, and those who were unemployed, underscoring the importance of targeting health interventions toward these vulnerable subgroups.

Although moderate levels of practice were reported, particularly in blood pressure and weight monitoring, behaviors directly linked to eye care, such as blood sugar control and regular eye examinations, were far less consistent. These patterns reflect a disconnect between general diabetes self-management and specific knowledge about its ocular complications.

Importantly, our study revealed statistically significant associations between KAP scores and multiple sociodemographic factors, including gender, education level, occupation, and duration of diabetes. These findings reinforce that DR screening adherence is not solely a matter of access but is influenced by broader social determinants of health, a point that should guide intervention strategies.

Socioeconomic disparities appear to play a critical role in shaping patients’ knowledge and engagement with DR care. In our sample, participants with higher education and active employment demonstrated significantly better knowledge and practice scores, whereas those with lower education or unemployment showed poorer awareness and less favorable screening behaviors. These findings align with national evidence indicating that health literacy and income inequality influence adherence to diabetes screening programs in Saudi Arabia^[^11^]^. Limited educational attainment may restrict understanding of DR’s asymptomatic nature, while occupational instability reduces healthcare access and prioritization. Addressing these structural barriers through targeted health education and integration of eye-screening reminders in primary care could mitigate inequities in early detection and reduce preventable vision loss.

Comparable international studies emphasize persistent barriers to DR screening and provide valuable implementation strategies. For instance, in England, 80% of individuals newly registered with the NHS Diabetic Eye Screening Program attended screening within 12 months; however, younger adults, socially deprived groups, and ethnic minorities exhibited significantly lower screening rates^[^12^]^.

Moreover, the Singapore Integrated Diabetic Retinopathy Program demonstrated cost savings of S$144 per person and high quality-adjusted life year by shifting from physician-based to technician-led tele-ophthalmology screening, supporting scalable primary-care integration^[^13^]^. Drawing on these models, implementing mobile-health reminders, automatic electronic health record prompts for clinicians, and co-located retinal imaging in primary-care clinics would be strategically appropriate for Saudi Arabia to enhance screening adherence and early DR detection.

### Public health and clinical implications

Our findings support a multi-pronged strategy to improve DR awareness and screening adherence:

*Enhance physician training and electronic health record prompts* to ensure DR screening is discussed and documented during diabetes follow-up visits.

*Develop culturally tailored educational materials* that address common misconceptions and use simple, locally-appropriate language.

*Leverage mobile health (mHealth) technologies*, such as SMS reminders or patient portals, to nudge patients toward annual screenings.

Strengthen referral linkages between primary care and *ophthalmology* through streamlined scheduling or in-clinic retinal photography.

Furthermore, the high prevalence of patients reporting laser treatment without routine prior screening suggests that many are being diagnosed only after advanced disease has developed. This reinforces the importance of shifting from reactive to proactive eye care in the T2DM population.

## Limitations

This study has several limitations. The use of convenience sampling may limit generalizability to all T2DM patients in Saudi Arabia. As this was a cross-sectional study, causal relationships between knowledge, attitudes, and screening practices could not be established; therefore, all reported associations should be interpreted cautiously. Self-reported responses are subject to recall and social desirability biases. Nevertheless, the large sample size and use of a validated instrument enhance the reliability of the findings and their relevance to real-world clinical settings.

## Conclusion

Although participants demonstrated a moderate level of knowledge (mean score = 1.77 ± 0.23), both attitudes (1.20 ± 0.20) and practices (1.53 ± 0.29) toward DR screening were suboptimal. Only 28.5% underwent an eye examination within the past year, and merely 10.5% recalled receiving physician advice for screening. These findings underscore the gap between awareness and behavior and highlight the urgent need to strengthen referral pathways, enhance patient–provider communication, and implement culturally tailored educational initiatives to improve early detection and reduce DR-related vision loss.

## Data Availability

The datasets generated and anlyzed during the current study are availble upon request.
